# Assessing receptiveness to change among primary healthcare providers by adopting the consolidated framework for implementation research (CFIR)

**DOI:** 10.1186/s12913-019-4312-x

**Published:** 2019-07-16

**Authors:** Lee Lan Low, Fathullah Iqbal Ab Rahim, Mohammad Zabri Johari, Zalilah Abdullah, Siti Hajar Abdul Aziz, Nur Ajeerah Suhaimi, Norrafizah Jaafar, Ainul Nadziha Mohd Hanafiah, Yuke Lin Kong, Siti Haniza Mahmud, Mohamad Zaidan Zulkepli, Komathi Perialathan, Norazlin Muharam, Nur Hani Zainudin, Zaikiah Mohd Zin, Norazilah Mohd Roslan, Tahir Aris, Shahnaz Murad

**Affiliations:** 1 Institute for Health Systems Research, National Institute of Health, Ministry of Health Malaysia, Block B2, No. 1, Jalan Setia Murni U13/52, Seksyen U13 Setia Alam, 40170 Shah Alam, Selangor Malaysia; 2 Institute for Behavioural Research, National Institute of Health, Ministry of Health Malaysia, Block B3, No. 1, Jalan Setia Murni U13/52, Seksyen U13 Setia Alam, 40170 Shah Alam, Selangor Malaysia; 3 Department of Statistics, Block C6, Complex C, Federal Government Administrative CentrE, 62514 Putrajaya, Malaysia; 4 Family Health Development Division, Putrajaya, Ministry of Health Malaysia, Block E1, E3, E7 & E10, Complex E, Federal Government Administrative Centre, 62590 Putrajaya, Malaysia; 5 Institute for Public Health, National Institute of Health, Ministry of Health Malaysia, Block B5, No. 1, Jalan Setia Murni U13/52, Seksyen U13 Setia Alam, 40170 Shah Alam, Selangor Malaysia; 6 Office of Deputy Director General of Health (Research and Technical Support), Ministry of Health Malaysia, Block E1, E3, E7 & E10, Complex E, Federal Government Administrative Centre, 62590 Putrajaya, Malaysia

**Keywords:** Readiness to change, Qualitative, Primary healthcare, Non-communicable diseases, CFIR

## Abstract

**Background:**

Amid the current burden of non-communicable (NCD) diseases in Malaysia, there is a growing demand for more efficient service delivery of primary healthcare. A complex intervention is proposed to improve NCD management in Malaysia. This exploratory study aimed to assess primary healthcare providers’ receptiveness towards change prior to implementation of the proposed complex intervention.

**Method:**

This study was conducted using an exploratory qualitative approach on purposely selected healthcare providers at primary healthcare clinics. Twenty focus group discussions and three in-depth interviews were conducted using a semi-structured interview guide. Consent was obtained prior to interviews and for audio-recordings. Interviews were transcribed verbatim and thematically analysed, guided by the Consolidated Framework for Implementation Research (CFIR), a framework comprised of five major domains promoting implementation theory development and verification across multiple contexts.

**Results:**

The study revealed via CFIR that most primary healthcare providers were receptive towards any proposed changes or intervention for the betterment of NCD care management. However, many challenges were outlined across four CFIR domains—intervention characteristics, outer setting, inner setting, and individual characteristics—that included perceived barriers to implementation. Perception of issues that triggered proposed changes reflected the current situation, including existing facilitating aspects that can support the implementation of any future intervention. The importance of strengthening the primary healthcare delivery system was also expressed.

**Conclusion:**

Understanding existing situations faced at the primary healthcare setting is imperative prior to implementation of any intervention. Healthcare providers’ receptiveness to change was explored, and using CFIR framework, challenges or perceived barriers among healthcare providers were identified. CFIR was able to outline the clinics’ setting, individual behaviour and external agency factors that have direct impact to the organisation. These are important indicators in ensuring feasibility, effectiveness and sustainability of any intervention, as well as future scalability considerations.

**Electronic supplementary material:**

The online version of this article (10.1186/s12913-019-4312-x) contains supplementary material, which is available to authorized users.

## Background

Improving performance and initiating broad scale changes at the healthcare organisational level often involve multiple or a combination of interventions that may be complex and multi-faceted and will require careful coordination at all levels, including specific contextual adaptations to the implementation [1, 2]. In Malaysia, the non-communicable disease (NCD) burden is growing at a very alarming rate, indicating the need for improvements in the healthcare system, especially at the primary healthcare level [3]. The Ministry of Health (MOH) embarked on a complex intervention at the primary healthcare setting, dubbed the Enhanced Primary Healthcare (EnPHC) initiative, which aimed at (1) delivering more effective and efficient healthcare services to NCD patients, and (2) improving patient experience and (3) health outcomes over a period of time [4].

The proposed EnPHC’s intervention components include establishment of community interventions through fostering population enrolment and population risk profiling for early NCD risk management and case detection, deployment of a two-tiered triaging system in primary healthcare clinics, introduction of the care coordinator role to improve the community engagement mechanisms in facilitating clinic appointments, treatment and medication, improvements in the NCD care management process and improvisations in the communication process for intra-clinic referrals and referrals between clinics and hospitals [5].

Before any new intervention is introduced, addressing organisational readiness and healthcare providers’ (HCPs) receptiveness to change is essential; it is a critical precursor for successful implementation of complex changes in a healthcare setting [6]. ‘Readiness for change’ (hereafter referred to as readiness) is a state of being both psychologically and behaviourally prepared to take action [6] to move into a new and different state [7]. ‘Openness to change’ (hereafter referred to as openness and is terminologically interchangeable with receptiveness) is willingness to accept change and have positive views of changes [8] in which personal resilience, information about changes, self-efficacy for coping with changes and participation in the change decision process were predictive of higher employee openness. The major distinction between readiness and openness is the knowledge about intended change outcome, which acts as a vital component in openness compared to readiness [9]. Thus, both organisational readiness and individual employees’ openness are important determinants of implementation success.

The Consolidated Framework of Implementation Research (CFIR) was selected for this study because it can be used flexibly across a wide range of applications at any implementation phase (pre, during, post) [10]. The meta-theoretical CFIR was developed based on a comprehensive review of literatures and models. It integrated multiple frameworks into one consolidated framework and comprised of influential pre-specified factors that are relevant to implementation studies [11, 12]. The four major domains of CFIR—intervention characteristics, outer setting, inner setting and characteristics of individuals [13] were used to practically guide the assessment of organisational readiness and employee openness, including both facilitators and barriers, as well as to facilitate the strategies for development of intervention materials and manage issues related to implementation readiness [12, 14, 15]. CFIR can be employed to assess pre-intervention or implementation information such as perceptions of implementers.

This study aimed to explore receptiveness to change among primary HCPs and other challenges at primary healthcare clinics prior to implementation of EnPHC. Data presented here were collected as part of a larger process evaluation of a complex intervention study. The findings will be used to improve the pre-designed intervention before it is scaled up nationwide.

## Methods

### Study design

This exploratory qualitative study consists of focus group discussions and in-depth interviews that were conducted in eight selected primary healthcare clinics within two states (Selangor and Johor) in Malaysia, which were the pilot sites for the EnPHC initiative. Four CFIR domains were used to assess the HCPs’ receptiveness to the proposed change.

### Study setting and participants

In Malaysia, public primary healthcare clinics provide primary access for curative, preventive and health promotion services to the community. The clinics are categorised according to the population size served and service availability [16]. Clinics typically consist of a multi-disciplinary team encompassing family medicine specialists (FMS) (in some clinics), general medical practitioners, and paramedics such as nurses and assistant medical officers, physiotherapists, nutritionists, dieticians and occupational therapists. The clinics were purposively sampled from the urban and rural strata, which served a range of 200 to 1000 patients on a daily basis depending on the type of clinics and locations. In all, 106 HCPs from various categories were included to achieve representation across all levels of staff. The focus group discussions were conducted according to their homogenous group to encourage open discussion and freedom of expression towards readiness to change. The participants may have possibly heard about the EnPHC initiative that was to be implemented, but they did not have exact information regarding the proposed interventions. Furthermore, the researchers did not reveal any information on the initiative to eliminate bias in perceptions across all participants, especially since the actual components of the proposed complex intervention package had yet to be finalised at the time of our data collection.

### Data collection

Data were collected between April and May 2017. Overall, 20 focus group discussion (FGD) sessions involving 40 health professionals and 66 paramedics were conducted; each session included six to eight participants. In-depth interviews (IDI) were also conducted with three FMS from selected clinics. The FMS is usually the highest-level authority in the clinic, hence, separate interview sessions were conducted to avoid uneasiness by other clinic members in expressing their views. Information saturation was achieved when no new information can be added to HCPs’ perception of change. For this study, saturation was achieved after 18 FGD whereby all of the identified domains did not have any new information. The additional two FGD were conducted as confirmatory measure to ensure no new information were found. All 106 participants were invited through their heads of clinics. Interviews were conducted at their workplaces (clinic) and during their preferred accommodated times. This approach was adopted to maximise participation and was found to be fruitful as there were no dropouts.

Prior to fieldwork, a comprehensive interview guide with semi-structured questions was developed to assist the interviewers. Topics included openness, perceptions and readiness to change, aside from other general questions such as service duration in the NCD field and the difficulties or challenges faced at their current workplaces. The interview guide underwent revision to include topics such as perception on the need for improvement in NCD care management and triggers for change (Aditional file 1).

The principle investigator (LLL) contacted all medical officers-in-charge of each study clinic, briefed them about the data collection process and scheduled the interview sessions. Eligible participants were briefed prior to their interviews about the study’s purpose, process, confidentiality assurance, voluntary nature of participation and freedom to withdraw from the study at any point in time. Participants were also given the chance to ask questions. Bilingual (English and Malay) participant information sheets were provided. Written consent and permission for audio recording were obtained from all participants prior to interview sessions.

The FGDs and IDIs were conducted by a minimum of two qualitative researchers, one as the moderator and another as the note-taker. All sessions were audio-recorded to ensure data collection accuracy. Each IDI lasted between 30 to 60 minutes, while the FGDs ranged between 60 to 90 minutes. Sessions were carried out either in mono-, bilingual or mixed language, depending on the comfort and fluency of participants and researchers.

### Analysis

Data transcription and analysis were carried out immediately after each interview. All interviews were transcribed verbatim into Microsoft Word and subsequently transferred to Nvivo11™ to facilitate data management and coding process. Interviews in Malay language were transcribed without translations, but coded in English. The selected quotes in Malay were translated into English for report writing and publication purposes.

An open coding approach was applied to the transcripts at the first order coding before subsequent grouping in second order coding. To imbue sense into the thematic codes, a framework analysis approach guided by CFIR was carried out. A codebook was developed by adopting from CFIR’s website prior to third order coding [17]. Initially, all 39 CFIR constructs were included, but the domain ‘Intervention Process’ was deemed irrelevant at the pre-implementation stage. Hence, only the four domains of ‘Intervention Characteristics’, ‘Outer Setting’, ‘Inner Setting’ and ‘Characteristics of Individual’ were used for analysis. Operational definitions for these constructs were developed with regard to EnPHC implementation to capture contextual factors that might influence implementation effectiveness. Coders are members of EnPHC-Process Evaluation team who are familiar with and understand that CFIR constructs were required to strictly adhere to the predetermined operational definition and apply codes without making inferences from the data. However, analysis was not limited only to CFIR constructs because flexibility to create new subdomains and categories that may arise from the data inductively was allowed. The coding process was done independently in pairs, during which the same transcript was read by two persons who coded it separately, followed by consensual validation among the researchers before codes and verbatim quotes were regrouped into major themes. The codebook was continuously revised throughout the data analysis process, during which additional subdomains and their definitions were added or removed.

## Results

This qualitative exploratory study involved participants from various levels and categories of HCPs. Table 1 presents participants’ demographics. In all, 106 HCPs in the selected primary healthcare clinics were recruited based on their availability and demographic characteristics. This was to obtain the maximum variation of characteristics of the clinics’ total make-ups. For the purpose of analysis, only four of the five relevant CFIR domains were used for this paper.

**Table 1:** Participants’ demographic in EnPHC pre-implementation study

Health care providers	No. of participants
Professional	
FMS	3^a^
Medical officer	25
Pharmacist	11
Paramedics^b^	
Nurse	30
Assistance medical officer	18
Clinical Support	
Nutritionist	1
Physiotherapist	3
Medical lab technologist	5
Radiologist	1
Assistance pharmacist	8
Others	1
total	106
Type of Public Primary Healthcare Clinics^c^	
Type 2	35
Type 3	14
Type 4	57

Note:

^a^In-depth Interview’s participants

^b^The Paramedics and Clinical Support groups shall be hereafter referred to as Paramedics in this paper

^c^Informational source on clinics type from Town Planning document [18]

### Identified barriers and facilitators based on CFIR constructs and sub-constructs

Table 2 summarises the details of 20 constructs and sub-constructs from the four selected CFIR domains that the HCPs perceived as positive or negative in their clinic environment and setting. The presence of positive perceptions was more likely to facilitate change in implementing new interventions. Negative presence was a perceived barrier and challenge for change implementation. During preliminary analysis, ‘non-compliance to appointment’ and ‘non-compliance to treatment management’ seemed to fit a new construct that was initially labelled as ‘Patient’s Behaviour’, which was not in the CFIR. After long deliberation, codes in this new construct were re-fitted into CFIR’s ‘Patients and Resources’ construct.

**Table 2:** Perception on change mapped on consolidated framework for implementation research domains

Domain	Construct	Sub-construct	Perception on change	
			Positive Presence	Negative Presence
Intervention Characteristic	Intervention source			
	Evidence strength & quality			
	Relative advantage			
	Adaptability			
	Trialability			
	Complexity			
	Design quality and packaging		**/**	
	Cost			
Outer Setting	Patient needs & resources		**/**	**/**
	Cosmopolitanism		**/**	**/**
	Peer pressure			
	External policies & incentives			**/**
Inner Setting	Structural characteristics			**/**
	Network & communications		**/**	
	Culture		**/**	**/**
	Implementation climate	Tension for change	**/**	
		Compatibility		**/**
		Relative priority	**/**	
		Incentives & rewards	**/**	
		Goals & feedback		
		Learning climate	**/**	
	Readiness for implementation	Leadership engagement	**/**	
		Available resources		**/**
		Access to knowledge & information	**/**	**/**
Characteristics of individuals	Knowledge & belief about the implementation		**/**	
	Self- efficacy		**/**	
	Individual stage of change		**/**	
	Individual identification with organisation		**/**	
	Other personal attributes		**/**	

Note: positive presence would facilitate the implementation of new intervention, negative presence as perceived barrier to change by study participants

Table 3 provides selected quotes from the participants’ view of barriers and facilitators for implementing a new intervention at primary healthcare. Several challenges were perceived as barriers for introducing a new intervention under the domains of intervention characteristics, outer and inner setting. Despite these challenges, HCPs expressed openness to any new intervention for enhancing the NCD care management. Their readiness to change was based on their experience in managing their clinics and their personal insight of their clinic settings that may facilitate the implementation of new intervention.

**Table 3:** Example quotes organised thematically and mapped to CFIR domain and construct base on barrier and facilitator

Domain	Construct	Barrier/facilitator	Quotes
Intervention Characteristic	Design Quality and Packaging	Perceived facilitators: dedicated team for NCD Management	If we are to truly focus on NCD services for patients, a dedicated team should be developed at the clinic. (paramedic, 34 years old)
Outer Setting	Patient Needs and Resources	Perceived barrier: non-compliance to appointment time	Most of our patients [here] are elderly. Their children will drop them off early in the morning before going off to work. They [elderly patients] will congregate [at the clinic] at the same time. We cannot implement the staggered-time appointment, even when we emphasised it. [That is why] the small OPD [outpatient department] area is congested [because] many patients are waiting there. (professional, 33 years old)
		Perceived barrier: patients’ beliefs	People always have this concept about KK [health clinic], it’s a place to take medicine and medical certificate [MC]. But they never thought of KK as a place [for] health [improvement] opportunity. (professional, 36 years old)
		Perceived facilitators: clinic location	Most patients love to come here [clinic] because it’s near to town and is easy since consultation, blood tests and analyses can be completed within one day. (professional, 49 years old)
	Cosmopolitanism	Perceived barrier: lack of inter-facility networking	Whenever patient [is] discharged from a hospital, they will bring a [referral] letter from the hospital to come to the clinic. That is the only thing [document]...we do not have any other way [to access information]. Sometimes, the letter might not complete, investigations done might not be there (professional, 28 years old)
		Perceived facilitators: relationship and networking with NGO and community	If that is true [collaborating with KOSPEN], it [will be] very helpful because we do not have [the] opportunity [to perform] screening. Sometimes, a patient, especially elderly, is afraid of the clinics [staff]. But if its KOSPEN, [patients] will come [for screening] (professional, 42 years old)
	External Policy and Incentives	Perceived barrier: top-down mandates with new unplanned programme	Sometimes the programmes [directives from top management] come suddenly and they want the result immediately...it is disruptive, given [short] period of time and we have to rush. (paramedic, 32 years old)
Inner Setting	Structural Characteristics	Perceived barrier: physical space constraints to deliver services	We are sharing room for fundus assessment with dental services. Because dental officers will come our clinic once a month. So, when there is an appointment for dental, the room becomes a dental room and will not be open for fundus assessment. So when a patient comes for a fundus appointment that coincidentally clashes with a dentist slot, his/her assessment will be postponed to another day. (paramedic, 27 years old)
	Networks and Communications	Perceived facilitators: Information sharing	If we need [something], we just can talk [share the information] …. If not, during the morning briefing, anything urgent we can discuss with the head of unit [about] any problem or current issues, and they will highlight [and] go through it. (professional, 56 years old)
	Culture	Perceived facilitators: teamwork	Relationship [between staff] is better, good, because we have no blocks [no barrier] here. I mean, the last time we have an OPD [outpatient department] wing and a MCH [Maternal Child Health] wing. Now, no [more]...we are all under one clinic. (professional, 55 years old)
		Perceived barrier: divided zone leads to silos working culture	We are divided into two zones/areas here [for family doctor concept]—a blue zone and a pink zone. Previously, there were no zones, only one area; we would automatically help each other. But when there are zones like this, there’s no initiative from staffs at the pink zone to help the blue zone or vice versa (paramedic, 31 years old)
	Implementation Climate i) Tension for Change	Perceived facilitators: increasing service demand	Increase in population... currently around 50 thousand. Before, there are around 20 thousand [patients]. The number grew a lot. (professional, 33 years old)
		Perceived facilitators: Standardisation of work process	Work process. The patient flow... That’s why things are not standardised. (laboratory technician, 36 years old)
		Perceived facilitators: Improving documentation process	For every single thing you have to documentation … documentation … documentation.... But you are doing the repetitive work...for the medical assistants or nurses every month, they have to repeatedly key-in all these work into their computers to send to the [state] office. (professional, 40 years old)
		Perceived facilitators: Improving consultation time	We don’t have enough time to counsel patients. Because when the number of patients increases, everyone wants to be fast and we are unable to focus. NCD has lots of factors [things to talk about] but we only have five minutes to counsel [patients]. (professional, 27 years old)
	Implementation Climate ii) Compatibility	Perceived barrier: lack of proper tracking mechanism	We have received referrals from KOSPEN, but the issue is patient does not come [for appointments] sometimes … there is no mechanism that states how we want to cater [to all referrals]. (Professional, 42 years old)
	Implementation Climate iii) Relative Priority	Perceived facilitators: accepting the concept family doctor	The same doctor [for a patient], the doctors will understand and be more familiar about their [patients’] health. (paramedic, 42 years old)
	Implementation Climate iv) Organisation Incentives and Rewards	Perceived facilitators: services performance recognition	I think it’s like a reward...we receive a plaque and the staffs were happy...we [our clinic] are the best maternal child health [performance] in this state; so we are proud. (professional, 42 years old)
	Implementation Climate v) Learning Climate	Perceived facilitators: avenue for discussion	He [medical officer in charge] is great. If anything goes wrong … he’ll call all of us [and] discuss. (paramedic, 32 years old)
	Readiness for Implementation i) Leadership Engagement	Perceived facilitators: involvement of leaders with implementation	They [leaders] are willing to look at it [problem] and try to solve [it]. They are very approachable. (professional, 30 years old)
	Readiness for Implementation ii) Available Resources	Perceived barrier: minimal manpower	We only have two staff at the community clinic, one working at the clinic and the other one does home visits. So the one who is out for home visit, even in an isolated area, will be working alone as we only have one transportation. (paramedic, 45 years old)
	Readiness for Implementation iii) Access to Knowledge and Information	Perceived facilitators: SOP and guidelines made available	Now there’s a SOP that everyone supposed to know. They should know what readings should be read; what to do about it … meaning there is already a guideline. It makes its easier to handle NCD cases. (paramedic, 45 years old)
		Perceived barrier: insufficient information to introduce new programme	An example of programmes that I would like to mention is the FDC [family doctor concept]—it was not proper, not clear. We forced ourselves to make it clear for us because we have to implement the program. (professional, 32 years old)
Characteristic of Individual	Knowledge and Beliefs	Perceived facilitators: regular updating knowledge	To me, [the proposed] EnPHC has many good sides. When we take good care of our patients, their health will improve. In the end, it will benefit us [providers] too, as indirectly, our patient load will be reduced. We think in future, the workload will be diminished. No more people will suffer from diabetes, high blood pressure. (paramedic, 45 years old)
	Self-efficacy	Perceived facilitators: own capability to perform	Why are we so eager about it? One, we have an indicator that needs to be pursued. The second one, one of the main duties of the assistance medical officer is a prevention programme. So, I take as my responsibility to make sure the programme is running. (paramedic, 30 years old)
	Individual Stage of Change	Perceived facilitators: embrace new ideas for improvement	I prefer when a new person comes to the clinic, he has a new idea. So, there may be new ways to implement....we can try and improve our programme. Because sometimes when an outsider comes in, we have new ideas. (paramedic, 30 years old)
	Individual Identification with Organisation	Perceived facilitators: work commitment	Like me, whenever I am given a task, I will do it. I would not decline [the task], but I may take some time. (paramedic, 41 years old)
	Other Personal Attributes	Perceived facilitators: professionalism and value	We actually want to give more [health education] to patients, but we don’t have enough [time] since many patients are waiting, so we have to make the consultation short...I will usually try to extend time [for] each session. I do not mind skipping my lunch, as long my patients are okay. (professional, 32 years old)

#### Intervention characteristics

Only one out of eight constructs for intervention characteristics emerged, with ‘Design Quality and Packaging’ highlighted as a facilitator. The availability of several activities (such as family doctor concept, community health empowerment programme and clinic appointment system) related to NCD management can facilitate the proposed plan for enhancing the NCD services and strengthen the suggestion for dedicated NCD care management teams in primary healthcare settings.

#### Outer setting

The outer setting has four constructs that refer to factors outside of the organisation. Three out of the four constructs (‘Patient Needs and Resources’, ‘Cosmopolitanism’ and ‘External Policies and Incentives’) were noted to have possible barriers. In contrast, clinic location and good networking with non-governmental organisations (NGOs) and community were seen as possible facilitators for implementing a new NCD programme. However, the perception of clinic location and accessibility as facilitators may vary depending on the facility location.

Patients’ non-compliance with appointment times was perceived as a barrier, especially among elderly patients who depend on their children for transportation. Consequently, the clinics’ current implementation of a time-slot appointment system (referred to as the ‘staggered appointment system’) did not run as intended. Additionally, HCPs noticed that the patients and the surrounding community have limited comprehension on the clinics’ role; they believed that patients perceived clinics as a curative centre rather than a place for learning about health. Professionals among the HCPs commonly recommended restructuring patients’ beliefs about the clinics’ role.

The construct of ‘Cosmopolitanism’ assessed the degree to which an organisation is networked with other external organisations. Lack of inter-health facility networking was perceived as a barrier in information transfer between primary healthcare clinics and hospitals, which affected patients’ care continuity. This challenge became more prominent when NCD patients who were discharged from hospitals were asked to continue their chronic care management in the primary care facilities within their localities. Nevertheless, the HCPs shared that the presence of good networking and relationships with other external organisations such as NGOs and other ministries were viewed as an advantage for interventions that require external support. For example, the community health empowerment programmes such as *Komuniti Sihat Perkasa Negara* (KOSPEN) [19], was viewed as a good platform to introduce any preventive or health education activities.

Another barrier perceived by the HCPs was under the construct for ‘External Policies and Incentives’. It is related to mandates from top management that could sometimes be a challenge, especially for ad hoc directives that require urgent/immediate action. This often affected task prioritisation over on-going programmes, which has a further negative impact when the clinic has existing issues with insufficient resources. The participants shared this dilemma because they were concerned that the proposed new intervention will be implemented as an additional directive without proper planning or realignment with existing activities.

#### Inner setting

This domain consisted of five constructs with nine sub-constructs within the organisational context. Several barriers and facilitators were raised within the primary healthcare clinics’ context that covered all five constructs. Under the construct of Structural ‘Characteristic’, the constraint of clinic physical space was a concern that the HCPs raised. Facility upgrade was deemed crucial, as well as clinic resources, which largely have remained similar to when the clinic was first set up. This would prove challenging especially with the ever-increasing patient attendance that which is due to local population growth around the clinic. Resource issues will be elaborated within the sub-construct of ‘Available Resources’ under the ‘Readiness for Implementation’ construct. Good networking and communication within the clinic facilitates internal information relay regarding clarifications and issues requiring attention. HCPs in clinics that initiated morning briefings felt that the routine communication method boosts their morale and spirit to work.

‘Culture’ as a construct within inner setting is an important component in assessing organisational receptiveness and openness to new intervention. HCPs expressed that the culture of teamwork and helping one another had been established within the clinic. HCPs also shared that the sense of belonging to the clinic and professionalism were critical foundations for a strong internal teamwork. However, they were concerned that certain clinics have the culture of working in silos. It does not happen in all clinics, but they think that the concern warrants ministerial-level attention before it gets worse.

Under the ‘Implementation Climate’ construct, issues on barriers and facilitators were identified under five sub-constructs: ‘Tension to Change’, ‘Compatibility’, ‘Relative Priority’, ‘Organisation Incentives and Rewards’, and ‘Learning Climate’.

The ‘Tension for Change’ sub-construct assessed the stakeholders’ perception of the current situation as intolerable or needing change [17]. HCPs reflected on issues that triggered change and the clinics’ current situation. However, certain clinics viewed the patient influx as an opportunity for early detection and screenings, which may be a facilitator for enhancing NCD care management. The increasing clinic attendance also raised concerns among the doctors, who felt less time was spent with patients so as to not cause longer waiting times for patients-in-queue, causing comprehensive assessment and health education to be delivered inadequately. Regarding issues of unstandardised and disorganised workflow, HCPs shared their perception of the issues, their suggestions and attempted solutions in previous programme implementations. However, HCPs identified aspects in the current setting that demanded change, required strengthening or were perceived as intolerable. With regards to documentation strengthening, data entry for routine monthly returns has been done manually instead of computerised, which led to ‘Tension for Change’.

In addition, the challenges under the ‘Compatibility’ construct were raised. Referrals received from other MOH community screening programmes such as KOSPEN facilitated the clinics’ community screening activity. It was noted that there were no proper patient tracking mechanism in place after patients have been screened for NCD, which hinders efforts to establish person-centred care. Those who have been identified with medium and high risk by the screening activities may default clinic appointments for further management, hence, the lack of tracking mechanism results in the at-risk population to be left untreated until they actually require treatment. Hence, HCPs perceived proper documentation and establishment of a robust tracking mechanism as crucial to achieve person-centred care.

Under the ‘Relative Priority’ construct, the practice of family doctor concept was found to be a positive asset that could facilitate the proposed EnPHC initiative. This concept established one doctor and a team attending to the same patient to ensure care continuity, allowing them to have a better understanding of their patients’ health status and treatment.

A conducive ‘Learning Climate’ (a sub-construct under the ‘Implementation Climate’ construct) within the clinic encouraged staff to contribute ideas and opinions for service delivery improvements. Many HCPs expressed this view during the exploration on readiness. Acknowledgement of the ideas and opinions, and establishing avenues for discussion by the leader were seen as factors that create positive clinic environment in problem-solving activities.

‘Readiness for Implementation’ is another construct within the ‘Inner Setting’ domain that was defined as immediate indicators of organisational commitment to implement a new intervention. One of its sub-constructs is ‘Leadership Engagement’, which emphasise the leader’s role in an organisation. Approachable and dedicated leaders were viewed as catalysts for a successful intervention in which they are able to comprehend the programmes’ goals and successfully relay information to their staff. In other words, clinic staffs perceived that leadership’s willingness to participate and solve daily clinic issues established a strong support system within the clinic, consequently promoting staffs’ openness and readiness for any challenge.

‘Available Resources’ is a sub-construct highlighted during the FGDs. Regardless of the HCP’s enthusiasm towards change, resource availability was critical in determining the level of staffs’ readiness. Resources covered a substantial range of aspects from operational components such as training and funding, to organisational components such as buildings, equipment and staffing. There was a near-consensus among all participants on the need for additional staff and upgraded facilities to implement any new programme or interventions. HCPs shared that logistics was a challenge because most clinics only have one official vehicle, which was always in use. This forced the clinic staff to resort to using their own transportation despite the rare reimbursement from the respective district health offices.

The sub-construct ‘Access to Knowledge and Information’ or ease of access to information within the primary healthcare clinic was not an issue because the guideline or procedure was always available for reference, especially for NCD care management. However, not all programmes introduced in the clinic came with full implementation guidelines. One clinic shared that the introduction of the family doctor concept in their clinic was not provided with sufficient information or guidelines for implementation.

#### Individual characteristics

Under the ‘Individual Characteristics’ domain, all five constructs were found to have a positive presence that could be facilitators. Implementers’ ‘Knowledge and Belief about the Implementation’ is a crucial construct because previous study reported change created uncertainties and fear of the impending unknown [7] under individual characteristics. However, in this study, the HCPs expressed their expectation of improved overall health outcomes among patients with the proposed new intervention for NCD management. The ‘Self-efficacy’ construct was another facilitator as HCPs concurred that staff capability to execute tasks for NCD management was crucial since they are the key personnel who will be involved directly with the proposed intervention.

‘Individual Stage of Change’ depicts the manner in which HCPs embrace and accept new ideas for improvement, which denotes readiness. Ready HCPs are often open to new ideas, which they view as key to improve existing programmes. This type of mental readiness allows acceptance of new or untested methods to be easily implemented versus a non-ready HCP who would openly resist changes. Meanwhile, HCPs identified ‘Individual Identification with Organisation’ as their commitment to implementing MOH-driven programmes. One participant declared that any task assigned is a responsibility that must be executed despite the time needed to figure things out.

Other personal attributes such as motivation and individual positive attitudes contributed towards acceptance of previously implemented programmes. Some HCPs strongly believed the best care for patients must be provided for their good health outcomes. This reflected their professionalism and value, which could be facilitators for implementing new programmes or interventions.

## Discussion

This exploratory study aimed to assess the organisational receptiveness to change among primary healthcare providers prior to implementation of new interventions. Key findings from this study will help guide policy-makers and future research. We found almost all constructs to have positive presence, which supports the justification to implement the proposed intervention and suggests a potential smooth implementation. Previous studies reported that readiness at the organisational level is important for any implementation of complex interventions and changes in the healthcare system [20–24].

Most of the negative presences or perceived barriers identified were within the construct of ‘Outer Setting’ and ‘Inner Setting’, partly due to external agents, physical structure, resources and culture, which appear to be common in any organisation. Organisational leaders often introduce goal-oriented system-wide changes in an effort to address the ever-changing population needs. When these changes are introduced, differences and conflicts between organisation leaders and members might arise that may eventually affect the success of the implementation of an intervention. Barriers or concerns raised by organisation members need to be addressed prior to the introduction of a new intervention. To resolve conflicts, organisation members (HCPs within the clinic), beliefs and comprehension about the changes need to be aligned with the organisation leaders for the changes to be implemented successfully [25].

Introduction of new interventions should be aligned with daily activities of the HCPs in the clinics to motivate HCP work values. HCPs believed the current health delivery system needs change, specifically the system and work process, to address the increasing demands of the population (such as insufficient consultation time). This issue should be incorporated when designing a new intervention that meets the needs of both HCPs and the service quality for the population. Proposed interventions such as standardising work processes or improvising patient flow in the clinic [26] seemed to be supported positively by HCPs. Given the limited resources in the primary healthcare setting, additional programmes or new interventions are often perceived as additional burdens to the clinic. Considering the limitation of public primary healthcare clinics to hire additional staff, organisation leaders need to review, strategise and maximise existing internal resources.

Collaboration between primary health clinics and hospitals is reflected in the ‘Cosmopolitanism’ construct in the CFIR framework. Poor communication and networking between these facilities were seen as barriers in our study. Establishing a patient referral tracking [27] and utilising technology for information sharing between two facilities [28] might possibly overcome the obstacles, especially since it affects patient outcomes. Additionally, ‘Patient Needs and Resources’ must also be integral to the implementation [29].

Other barriers were collective attitudes and perspectives of those within the organisation such as physical structure limitations and the organisation’s work culture. Space constraints in an old building can act as a barrier for new interventions that require space. Organisation leaders who can provide flexibility in task execution and flexible organisation policies may reinforce staff’s readiness [30]. Establishment of teamwork and trust among team members also encourages the perception of readiness among the staff [30], and silo-based thinking within the organisation also needs to be addressed. Silos were unintentionally created as staff members focused on fulfilling the task at hand instead of achieving the overall outcome for the organisation, which is further disaggregated by the assigned zone, job types or job functions. Studies have addressed the silo working culture by establishing common performance goals, clear communication regarding vision [31], using a system thinking approach and encouraging communities of practice [32] and inter-professional team-based training that help improve clinic health delivery services [33]. Those are important strategies that the organisation leaders could explore prior to implementing the proposed interventions.

‘Learning Climate’ determines the openness of the leaders’ fallibility and willingness to embrace teamwork, feedback and input, thereby making team members feel needed, essential and valued [34]. This was supported by the satisfaction achieved when their feedback and suggestions seem to be accepted by their superiors. Sharing and engaging staffs regarding the vision and objectives of any new intervention would also most likely be welcomed by the organisation members. Leaders who engage their organisations by encouraging and influencing their staffs in achieving learning and operation-oriented goals might promote creative decisions and surrounding environments that are both stimulating and caring [35], all of which facilitate employees’ readiness towards change. Therefore, leadership and two-way communication play important roles in strengthening the organisation’s commitment.

To effectively implement the change and generate anticipated benefits, composed and coordinated behaviour of organisational members is crucial [6]. This statement aligns with our findings that all constructs under ‘Individual Characteristics’ showed positivity towards change among HCPs. Individual characteristics and behaviours are critical in influencing organisational outcomes and thus, are closely associated with organisational culture [36–38], which is defined as norms, values and basic assumptions of a given organisation [13]. If the characteristics of organisational culture are unclear and poorly communicated, the employees’ composed perspectives and their behaviours will be inconsistent and will become a challenge for readiness [39]. However, we found that this was not the case for the HCPs in our study, as the interviews indicate the organisational culture leaned towards positive impressions.

### Strengths and limitations

The Ministry of Health Malaysia plans to implement the EnPHC initiative, a complex intervention package, to improve NCD health service delivery in primary healthcare clinics. Findings from this exploratory study provide the baseline information on the perception of HCPs of various categories about their day-to-day clinic activities and local settings. Insights obtained from the findings will help programme planners and stakeholders plan the intervention deployment strategies. We found that applying the CFIR for formative evaluation provided better understanding of the components of outer and inner settings, as well as individual characteristics. Findings that were explored using the CFIR constructs also established a contextual knowledge platform that can be applied to other primary healthcare settings in the future. The focus group discussions and in-depth interviews allowed for deep understanding of the perceptions and local issues HCPs encountered in the clinic setting.

There were two limitations in our study. Firstly, the CFIR constructs were only applied later for analysis and were not used during the development of interview guidelines. Nevertheless, comprehensiveness of analysis guided by CFIR facilitates exploration of data from different dimension. Secondly, this study adopted only qualitative approach. Although qualitative data can provide rich information, quantitative measurement for the seriousness and severity of issues raised was not performed in the study. Future study, possibly using mixed method approach of a larger scale for an additional structured questionnaire on an item-scale (Likert for example) assessing the key readiness question in an indirect manner could explore perception as a cross-check measure.

## Conclusion

Understanding existing situations faced at the primary healthcare setting is imperative and HCPs receptiveness to change prior to implementation of any intervention is crucial. The CFIR was found to be an invaluable framework to assess the baseline (pre-intervention) perceptions of HCPs prior to the implementation of the EnPHC initiative. By using the CFIR constructs, the findings from this study identified human resources as the critical success factor for implementation. Most clinic staffs appeared to be receptive and eager for the proposed EnPHC changes in NCD patient management along with good governance by leaders that will ensure success in an organisation with sufficient manpower. These aspects are favourable predetermining factors for success.

However, as our study findings have shown, there are potential barriers with regards to resources. If EnPHC initiative is implemented, human and infrastructure resources, will be causes for concern especially because staffs have voiced their uneasiness about the patient load and the clinics’ facilities in the current Malaysia healthcare system (at the clinic level). In addition, there may also be cultural and local adaptations of the implementation in view of the variances of clinic setting e.g. level of engagement with KOSPEN and the perceived community mind-set e.g. non-compliance to clinic appointment issues. Given the top-down model of Malaysia’s healthcare delivery system, it is important for the programme planners and coordinators to have a careful assessment of the resources throughout this ‘pilot’ implementation, because HCPs are the key working gears for EnPHC’s successful implementation.

## Additional file

Additional file 1: Semi-structure questions for Focus Group Discussion/In-Depth Interview. (DOCX 72 kb)

## Abbreviations

CFIR: Consolidated Framework for Implementation Research
EnPHC: Enhanced Primary Healthcare
FMS: Family Medicine Specialist
HCP: Healthcare provider
KOSPEN: *Komuniti Sihat Perkasa Negara*
NCD: Non-communicable disease
