# Supplementary material for: Assessing receptiveness to change among primary healthcare providers by adopting the consolidated framework for implementation research (CFIR)
Source: BMC Health Serv Res. 2019 Jul 16;19:497. doi: 10.1186/s12913-019-4312-x (PMC6636000; doi:10.1186/s12913-019-4312-x)
Supplement: Supplementary file 1 — Semi-structure questions for Focus Group Discussion/In-Depth Interview. (DOCX 72 kb) [file 12913_2019_4312_MOESM1_ESM.docx]

**REVISED version 28 April 2017 (in blue color font as in the boxes)**

**Semi-structure questions for FOCUS GROUP DISCUSSION/IN-DEPTH INTERVIEW**

Target group: **Health Scare Providers (Control & Intervention)**

**General Questions**

1. How long have you worked in the NCD field/team?
2. Can you share any difficulties /challenges in your current work?

*Probe for details in difficulties and challenges.*

*How did you overcome the difficulties/challenges*?

**Openness to Changes**

1. Do you think that changes are necessary to improve your work in providing better NCD service at your workplace? Why the changes are necessary?

If participants share/complain issue related to their current work/program/clinic environment then can probe

- What are the issue?
- HOW that issue affected their current work/clinic environment
- What STRATEGIES been implemented to overcome that issue?

IF NOT, then ask if you have any suggestion or plan to ‘solve’ that issue?

**Changes and Relative Advantageous**

1. From your opinion, can you share whether if there is the change, can the change bring more:

- Benefit (positive/advantageous), *Probe for details -*  WHY?
- Disadvantage (negative impact), *Probe for details -* WHY?

*Probe for variations in answers by including or excluding the feedback given*:

| - Manpower (workforce numbers) - Facilities (equipment/computers) - Technology (new but unavailable tech at workplace) | - Structure (systemic/workflow) - Leadership (supportive/non-supportive leaders) - Teamwork (team player) |
| --- | --- |

*Avoid leading towards the items above but try to get participants to explain all the above.*

**Adoption of Changes(Time)**

1. Based on your past experience of implementing changes, how long would you think your suggested changes would be implemented effectively?

*Probe on each suggested changes*.

Please explained more what happened in the process?

**Resistance to Changes (Including Complexity, Compatibility &Trialability)**

1. If there is the change in your clinic,

- Do you anticipate **any barriers/difficulties** lead to change?
- Base on your clinic ‘environment’, what are the **enabler (facilitator)** factors can lead to smooth in change?
- Probe in term of setting, manpower, environment, leadership, teamwork
- Support (from who that they perceived is important, e.g. doctor in charge, PKD, JKN? MOH?)
- Clear guideline/instruction?
- How do you (and your colleague) accept new changes when it implemented?

Probe on teamwork, leadership and exclude existing facilities, focus on possible adaptation of new intervention

- Can you explain how the changes can improve the NCD service at your place?

*Probe on teamwork, leadership issues and exclude existing facilities; focus on possible adaptation of new intervention*.

1. Is there anything we should talk about, or any additional information we have not discussed?
